# Re-assessing causality between energy consumption and economic growth

**DOI:** 10.1371/journal.pone.0205671

**Published:** 2018-11-12

**Authors:** Atanu Ghoshray, Yurena Mendoza, Mercedes Monfort, Javier Ordoñez

**Affiliations:** 1 Newcastle University, Newcastle, United Kingdom; 2 Universidad Isabel I. Calle de Fernán González, Burgos, Spain; 3 IEI and Universitat Jaume I, Campus del Riu Sec, Castellón, Spain; Universidad de Castilla-La Mancha, SPAIN

## Abstract

The energy consumption-growth nexus has been widely studied in the empirical literature, though results have been inconclusive regarding the direction, or even the existence, of causality. These inconsistent results can be explained by two important limitations of the literature. First, the use of bivariate models, which fail to detect more complex causal relations, or the ad hoc approach to selecting variables in a multivariate framework; and, second, the use of linear causal models, which are unable to capture more complex nonlinear causal relationships. In this paper, we aim to overcome both limitations by analysing the energy consumption-growth nexus using a Flexible Fourier form due to Enders and Jones (2016). The analysis focuses on the US over the period 1949 to 2014. From our results we can conclude that, where the linear methodology supports the neutrality hypothesis (no causality between energy consumption and growth), the Flexible Fourier form points to the existence of causality from energy consumption to growth. This is contrary to the linear analysis, suggesting that lowering energy consumption would adversely affect US economic growth. Thus, by employing the Flexible Fourier form we find the conclusions can be quite different.

## Introduction

It has been argued that economic growth may exhaust resources and cause environmental degradation [1], compromising future growth. The fear that today's growth can cause other significant economic problems, especially for future generations, has propelled sustainable growth to the top of the political agenda for the vast majority of developed countries. Sustainability will depend on how the substitutability or complementarity between energy and production factors and the interplay with technical progress and productivity, impact economic growth. Consequently, and with the ultimate aim of assessing whether sustainable growth can be achieved, the causal relationship between economic growth and energy consumption has been widely debated in empirical studies. The energy-growth nexus has important policy implications. If increased energy consumption causes economic growth, sustainability can only be achieved by ensuring access to a cheap, safe, environmentally-friendly energy supply. Alternatively, if economic growth causes increased energy demand, the challenge is to reduce energy demand through market-oriented policies and regulatory instruments. A review of previous studies on growth-energy consumption using vector autoregressive (hereafter VAR) methodology is presented in Table 1. Till date, from Table 1, the results are inconsistent on the direction of causality between energy consumption and economic growth and even about the existence of causality [2–4]. Several factors lie behind these conflicting results. First, the data used in previous studies include different countries and time periods. Secondly, there are also differences in the variable selection; for example, most studies use aggregate energy consumption data, whereas a number of others examine various disaggregated measures. Thirdly, the studies also differ in terms of the econometric methodology.

**Table 1:** Summary of studies on growth-energy consumption nexus.

YEAR	AUTHOR	METHODOLOGY	VARIABLES/COUNTRIES/SPAN	RESULTS
2017	[5]	STIRPAT model	Three different level of urbanization (as a growth indicator), energy intensity,266 prefecture-level, 2000–2010 period	Positive impact for all groups of urbanization on energy consumption
2017	[6]	Panel Vector Autoregressive (PVAR) and impulse response function.	Energy consumption, economic growth and CO_2_ emissions 106 countries classified by different income groups over the period 1971–2011.	*Feedback Hypothesis*: EC → EG EG → EC
2016	[7]	Using the neoclassical Solow growth framework test Granger Causality.	Energy consumption and economic growth in Vietnam for the 1971–2011 period.	*Growth Hypothesis*: EC → EG
2016	[8]	Panel Cointegration and Vector error-correction model (VECM)	Energy consumption, economic growth and CO_2_ emissions. 188 countries for the periods 1993–2010.	EC negatively affects EG World as a whole: *Neutrality Hypothesis* EC not affects EG In developing countries.
2016	[9]	VAR time-varying	Energy consumption, economic growth and CO_2_ emissions. The sampled countries are Bangladesh, Egypt, Indonesia, Iran, Mexico, Nigeria, Pakistan, the Philippines, Turkey, South Korea, and Vietnam over the period 1972–2013.	*Conservation hypothesis*: EG → EC in the Philippines, Turkey, and Vietnam *Feedback hypothesis* EC → EG EG → EC In South Korea
2016	[10]	Panel Vector Autoregressive (PVAR) in a generalized method of moments (GMM) framework	Energy consumption and economic growth, and how democracy moderates this relationship using panel data of 16 sub-Saharan African (SSA) countries for the period 1971–2013.	*Feedback Hypothesis* EC → EG EG → EC
2015	[11].	Time-varying (TV) causalities	G7 (excluding Germany). 1960–2010	*Feedback Hypothesis* EC → EG EG → EC For Japan. *Conservation hypothesis*: EG → EC in Italy.*Growth Hypothesis*: EC → EG Canada Neutrality hypothesis:France, United States and UK.
2015	[12]	Johansen co-integration test and Granger causality test	Countries: Indonesia, Malaysia, Thailand, Singapore, and the Philippines. 1980–2012	EC affect to EG for almost all ASEAN-5 countries.
2015	[13]	VECM.	Energy consumption and industrial production in Tunisia for the period 1980–2007.	*Neutrality hypothesis*:At aggregate level.
2014	[14]	Meta-analysis of 158 studies in Energy-GDP nexus.	1978–2011	*Results of causality direction is forcefully depend on analysis and econometric methods*.
2013	[15]	VECM	Economic growth, energy consumption and financial development for Malaysia. From 1971 to 2009.	*The results suggest that energy consumption is influenced by economic growth and financial development*, *both in the short and the long run*
2012	[16]	VECM	energy consumption, electricity consumption, carbon emissions and economic growth in Bangladesh. 1972–2006.	EC→ Economic Growth in the short and the long run

Note: VECM is a reparameterized VAR model with integrated variables.

The majority of the early studies confine the analysis to the bivariate causal relationship between energy consumption and real output. A common problem with the bivariate model specification is the possibility of omitted variable bias [17,18], with the consequent loss of information that may be relevant in determining the direction of causality. Over recent years, several authors have attempted to overcome this problem by including additional variables in the causal analysis of the relationship between energy consumption and growth. However, as pointed out by [19], these additional variables have been selected on a rather ad hoc basis and the results on causality may be influenced by variable selection bias. Related to this problem of selection bias and variable omission, [20] point out that the absence of a prior theoretical model may cause the causality test to deliver mixed results. To address the lack of statistical motivation when choosing the control variables for the causal analysis, [19] apply a robust Bayesian probabilistic model to select the explanatory variables to be considered in the causal analysis of the relationship between energy consumption and economic growth. This approach allows for the evaluation of the posterior probability of including in the model a control variable selected from a large group of possible candidates; to the best of our knowledge, it is the first time in the literature that a robust variable selection method has been applied for this purpose. [19] use this method to select control variables for the analysis of the relationship between energy consumption and growth in the US from 1949 and 2010, for both aggregated and disaggregated data.

Most empirical studies test for causality in a linear framework (for example using Granger-Sims causality tests and/or unit root and cointegration techniques with either time series or panel data), neglecting the possibility of nonlinear causality. However, given the growing evidence of the presence of possible nonlinearity in several macroeconomic time series which could be caused as a result of several structural breaks, there has been an increasing reliance on nonlinear techniques that could capture causal relations between such variables. Some authors argue that the linear approach to causality testing is limited in its capacity to detect certain kinds of nonlinear causal relationships and so recommend the use of nonlinear techniques [21–23].

While the linear VAR model has advantages in incorporating a large number of variables to be analyzed for Granger causality, there remain limitations, particularly relating to the underlying characteristics of the variables chosen in the model. The variables chosen in the present study are subject to structural breaks as documented by various studies. For example, [24] conclude structural breaks in oil prices, [25] find evidence of structural breaks in energy consumption and [26] conclude the presence of breaks in economic growth. Taking account of the recent studies in the area of energy consumption-economic growth nexus, the VAR modelling approach remains popular. However, as shown by [27] it is not straightforward to control for breaks in a VAR since a break in one variable will manifest itself in other variables of the VAR model, leading to model misspecification [28]. Accordingly, we propose to adopt a Flexible Fourier Form VAR (FFF-VAR) framework that allows for smooth breaks that increase the power and size properties of the model.

Therefore, our contribution to the extant literature is to add new findings to the energy consumption-economic growth literature using an alternative modelling approach. We test for causality between energy consumption and economic growth in the US in a multivariate framework, including variables such as those with the highest posterior probability of inclusion according to the results reported by [19], and at the same time, recognise the presence of the variables included in the VAR model to contain structural breaks and thereby choose an appropriate specification, the FFF-VAR to test for causality between the variables. This approach would be more conducive for the type of variables employed given the possibility of several gradual breaks which can be approximated by smooth breaks couched in the FFF-VAR model.

The remainder of this paper is organized as follows: in the next section, we discuss why it is important to consider nonlinearities when analysing the energy-growth nexus; we explain the econometric methodology in section 3 and present the results of our analysis in section 4. Finally, we outline our conclusions in section 5.

## Nonlinearities and the energy-growth nexus

In addition to the selection of relevant variables prior to the study of causality, as stated above, another potential cause of ambiguity in the empirical results on causality is the selection of the functional form of the test. The importance of not neglecting the nonlinearity in energy studies has been widely discussed in the literature. Table 2 summarizes the reasons suggested in the energy literature that motivates the use of a nonlinear framework. Specifically, [29] conclude that “Due to the influences of economic cycle fluctuations, macroeconomic policies, international oil price fluctuations, technological progress, and industrial adjustment, there may be a nonlinear relationship among economic growth, energy consumption, and CO2 emission” (pp. 1153).

**Table 2:** Literature review on nonlinearities in energy studies.

1. [29]	“Due to the influences of economic cycle fluctuations, macroeconomic policies, international oil price fluctuations, technological progress, and industrial adjustment, there may be a nonlinear relationship among economic growth, energy consumption, and CO2 emission.” (p.1153)
2. [30]	“The large number of nonlinear relationships embodied in economic variables have largely been ignored (Aderson et al., 2015). Granger (1988) pointed out that the world is almost certainly constituted by nonlinear relationships”.
3. [31]	“We employ a nonlinear panel smooth transition vector error correction model to recognize the possibility of regime shifts with respect to the determinants of renewable energy consumption”
4. [32]	“The effects of oil prices can be asymmetric, nonlinear and sensitive…For example, Hamilton (1983) shows that rising oil prices are responsible for nine out of ten of the U.S. recessions since the SecondWorld War. Zhang (2008) employs a nonlinear model to investigate the relationship between oil-price shock and economic growth in Japan, and shows the existence of nonlinearities and asymmetric linkages between the two variables studied. Lardic and Mignon (2008) reach the same conclusion for other developed economies from an asymmetric cointegration approach.”
5. [33]	“…exists a threshold effect between the two variables: different levels of economic growth bear different impacts on oil CO2 emissions”
6. [34]	“Economic events and regime changes such as changes in economic environment, changes in energy policy and fluctuations in energy price can cause structure changes in the pattern of energy consumption”

Despite the importance of nonlinearities in energy economics, the visibility of nonlinear econometric methodologies is surprisingly almost non-existent. For example, in a survey of more than fifty studies in the energy literature by [35] only one paper due to [34], consider a nonlinear functional form. Similarly, a survey by [36] cites a single paper by [31], that uses a nonlinear methodology in the energy-environment-growth nexus analysis. From the extant literature discussed above, both the empirical and the theoretical studies suggest several reasons to expect nonlinear behavior in the relationship between growth and energy consumption. The main arguments are:

### a) Energy prices cause different consumption levels

Historical events suggest that a significant and persistent increase in energy prices over time is usually followed by a downward adjustment of economic growth [37]. However, this adjustment is not instantaneous; there is a delay between the rise in prices and the fall in the level of production. After a time lag, this economic contraction causes a lower level of consumption, which is likely to be maintained until there is a significant change in energy prices, especially the case of oil.

It is important to note that the nonlinear pattern of energy prices could be reflected in the energy consumption-growth ratio, since energy prices may be the cause of certain contractions and expansions in growth, consequently resulting in different levels of energy consumption. The structural breaks in energy prices renders the linear framework unsuitable for capturing the dynamics of this relationship.

### b) Pollution haven hypothesis and porter hypothesis

More stringent environmental regulations increase competitive pressure, especially for those firms operating in the most polluting activities. Companies have a number of ways in which to adapt to regulations: first, they can buy emissions rights in order to continue consuming similar levels of energy; second, they can limit their consumption by producing less; a third alternative (called the Porter Hypothesis) is to invest in clean, efficient technologies that enable them to adapt to regulations while simultaneously boosting their competitiveness; or, fourth, they can move to countries with lax environmental regulations. This last strategy is known as the Pollution Haven Hypothesis (PHH), which states that companies in countries forced to comply with strict environmental regulations may eventually relocate to countries with weaker environmental laws.

According to the PHH, emissions in countries subject to regulatory pressure may decrease as a consequence of tightening environmental regulations. Nevertheless, it is unlikely that companies would all of a sudden "migrate" in response to the new regulatory framework. Instead, one would expect to find a gradual change in the deterministic structure of the relationship. On the other hand, the Porter Hypothesis holds that firms will introduce changes in production in order to comply with strict environmental policies and in an attempt to be more efficient and innovative. These changes will in turn affect energy consumption. Structural changes such as these are the result of progressive investment in cleaner, more efficient technologies. Therefore, models that allow for small but several breaks that can be approximated by smooth changes seem more suitable than linear models when it comes to capturing the effects envisaged by the Porter Hypothesis.

### c) Changes in sectoral specialization

Changes in the deterministic structure of the energy consumption-growth relationship can also be explained by the changes in the relative contribution different sectors make to GDP as a country experiences economic growth. There is a shift in the early stages of industrialization whereby sectors such as agriculture become less important than manufacturing; while in more advanced stages of development, manufacturing and other consumer goods sectors are replaced by the lower-consumption services sector. This undoubtedly creates a structural change in energy consumption that linear tests may be unable to capture.

### d) The environment as a luxury good

The Environmental Kuznets Curve (EKC) describes the change in a country’s emission levels over time as a result of its economic growth. In the early stages of industrialization, energy consumption rises sharply in countries that do not prioritize environmental degradation control. When countries reach a critical income level, their priorities switch to environmental protection, leading to changes in the energy regime.

To sum up, there are several possible reasons for the existence of nonlinearities in the energy consumption-economic growth relationship. Nevertheless, most studies analyze the energy consumption-economic growth relationship using a linear framework, which for reasons discussed earlier, are restrictive. Given the limitations, the most appropriate models for capturing a possible causality relationship between energy consumption and growth would be those that can approximate the small but several structural breaks that are likely to plague the variables chosen in this study. For the sake of comparison, we estimate both linear and non-linear models.

## Methodology

It is not unusual to find economic variables that contain multiple structural breaks. A vast plethora of studies have been put forward that test for structural breaks in individual time series variables. However, when the variables are couched in to a VAR model, this leads to a serious problem. For example, if there are structural breaks in just one variable in the VAR model, that can induce structural shifts in the other variables included in the model [28]. The problem is exacerbated in the VAR model as the breaks in the single variable affect other variables with a lag. [28] address this problem by building on the FFF-VAR model allowing for the Flexible Fourier Form to deal with possible multiple smooth shifts in the data.

To this end, [28] employ a variant of the Flexible Fourier Form due to [38] to deal with the possibility of multiple structural breaks in the variables included in the VAR model. To describe the method, consider the deterministic portion of one variable in the VAR to contain multiple structural breaks. This can be expressed as the deterministic part *d*_*it*_, of the equation for variable *y*_*it*_, given by:

$$d_{it}=\alpha_{i0}+\alpha_{i1}d_{1t}+\alpha_{i2}d_{2t}+\cdots +\alpha_{im}d_{mt}$$ (1)

where *d*_*it*_ represents the potential smooth functions over time, the parameters *α*_*ij*_(*j* = 1,2,…,*m*) indicate the size of break *j* on variable *i*; and *m* denotes the number of breaks in variable *i*.

If the breaks are sharp, then Heaviside Indicators could be employed such that *α*_*jt*_ = 1, if *t* > *t*_*j*_ and *α*_*jt*_ = 0 otherwise. However, if there are several breaks and they tend to be small, then the Flexible Fourier Form would be more appropriate that allows *α*_*jt*_ to be smooth functions over time. The procedure proposed by [28] represents the deterministic portion (*d*_*it*_) of the variable (*y*_*it*_) to be given by:

$$d_{it}=\delta_{i0}+\sum_{k=1}^{n}\phi_{ik}sin(2\pi kt/T)+\sum_{k=1}^{n}\psi_{ik}cos(2\pi kt/T)$$ (2)

This deterministic form is particularly useful in capturing the nature of the time series process that contains several small structural breaks with the help of low frequency components. In a way, the choice of the appropriate frequencies to include into the Flexible Fourier form controls for the structural breaks in the data. A test for nonlinearity can be conducted by performing a simple F-test for the exclusion restriction that all values of *ϕ*_*ik*_ = *ψ*_*ik*_ = 0 in (2). This is possible as [39] show that the *ϕ*_*ik*_ and *ψ*_*ik*_ in (2) have multivariate normal distributions. An advantage of the Flexible Fourier form is that it can mimic the nature of the breaks without any knowledge of the magnitude, location and the number of break dates. Besides, the Fourier approximation works for structural breaks which can be of either the innovational outlier or the additive outlier type.

Before proceeding to estimate a FFF-VAR model, it is necessary to test for stationarity of the variables included in the model. [40] have put forward an appropriate LM based unit root test that includes trigonometric components. The procedure involves estimating the following regression on an individual time series *y*_*t*_:

$${\Delta y}_{t}=c_{0}+\sum_{k=1}^{n}g_{k}sin(2\pi kt/T)+\sum_{k=1}^{n}h_{k}cos(2\pi kt/T)+v_{t}$$ (3)

Then using the estimates from the regression given by (3), the following regression is carried out:

$${\tilde{S}}_{t}=y_{t}-{\hat{c}}_{0}t-\sum_{k=1}^{n}{\hat{g}}_{k}sin(2\pi kt/T)+\sum_{k=1}^{n}{\hat{h}}_{k}cos(2\pi kt/T)$$ (4)

where ${\tilde{S}}_{t}$ is the detrended series and *k* represents the frequency selected for the approximation. The unit root test is carried out by estimating the following regression:

$${\Delta y}_{t}=\theta {\tilde{S}}_{t-1}+\lambda_{0}+\sum_{k=1}^{n}\lambda_{1i}\Delta sin(2\pi kt/T)+\sum_{k=1}^{n}\lambda_{2i}\Delta cos(2\pi kt/T)+\varepsilon_{t}$$ (5)

The null hypothesis of a unit root is given by *H*_0_:(*θ* = 0) and is tested using a Lagrange Multiplier (LM) test statistic given by *τ*_*LM*_. If the null hypothesis is rejected we can conclude that the data series is stationary. In the case of serially correlated errors, lagged values of Δ*y*_*t*_ are added to the regression so that the residuals are white noise. Given the limitation of the number of observations that we have, we choose to set *n* = *k* = 1. As emphasised by [40], a fourier form using *k* = 1 can serve as a reasonable approximation to breaks of unknown form.

If the flexible fourier form unit root tests lead us to conclude that the variables are stationary, then the variables are included in level form in the FFF-VAR model. For variables that are found to be integrated, they are differenced to be made stationary. The variables are stacked in a vector **z**′_*t*_ and the linear VAR would take the following form:

$$z_{t}=A_{0}+\sum_{i=1}^{l}A_{i}z_{t-i}+e_{t}$$ (6)

where **A**_0_ is a vector of intercepts, **A**_*i*_ a coefficient matrix while **e**_*t*_ is a vector of error terms. The lag length *l* is chosen according to the Akaike Information Criterion (AIC). We find the same lag length using the Bayesian Information Criterion (BIC). In the case of the Flexible Fourier Form, the VAR model is estimated as:

$$z_{t}=A_{0}(t)+\sum_{i=1}^{l}A_{i}z_{t-i}+e_{t}$$ (7)

where **A**_0_(*t*) = [*δ*_1_(*t*),*δ*_2_(*t*),*δ*_3_(*t*)]′

And each intercept *δ*_*i*_(*t*) depends on the *n* Fourier frequencies such that:

$$\delta_{i}(t)=a_{i}+b_{i}t+\sum_{k=1}^{n}a_{ik}sin(2\pi kt/T)+\sum_{k=1}^{n}a_{ik}cos(2\pi kt/T)$$ (8)

The FFF-VAR model has good size and power properties when testing for smooth structural changes in a VAR(1). In particular, with multiple structural breaks not particularly accounted for, the Granger causality tests tend to have poor size properties. The application of the FFF-VAR model to data that may contain multiple structural breaks, obviates these problems leading towards more reliable results.

## Data, results and discussion

In this section, we investigate the dynamic relationship between energy consumption and growth in the US. Making use of a Flexible Fourier form due to [28], we use causality analysis. As mentioned earlier, [19] develop a Bayesian model to select the variables with the highest posterior probability of explaining US growth; they consequently choose energy consumption (EC), public spending (SPE) and the oil price (OP) as covariates. Growth has been extracted from the US Bureau of Economic Analysis (http://www.bea.gov/) and is measured in million dollars as the ratio between Value Added (VA) and the the Value Added Price Index. Energy Consumption (EC), is measured in billion BTU, and has been obtained from US Energy Information Administration (http://www.eia.gov/). Oil Price (OP) corresponds to real oil prices in dollars per barrel, and has been obtained from InflationData.com (http://inflationdata.com/ Inflation/Inflation_Rate/ Historical_Oil_Prices_Table.asp), Public Spending (SPE) is measured as total real spending by the Government in million dollars and has been taken from (http://www.usgovernmentspending.com/spending_chart_1940_2017USk_13s1li011mcn_F0.

Clearly, excluding the oil price from the analysis of the causal link between energy consumption and growth may cause misleading results, to the extent that energy consumption and oil prices are connected. The inclusion of public spending allows us to control for other demand-side factors in the US growth.

**Fig 1:**
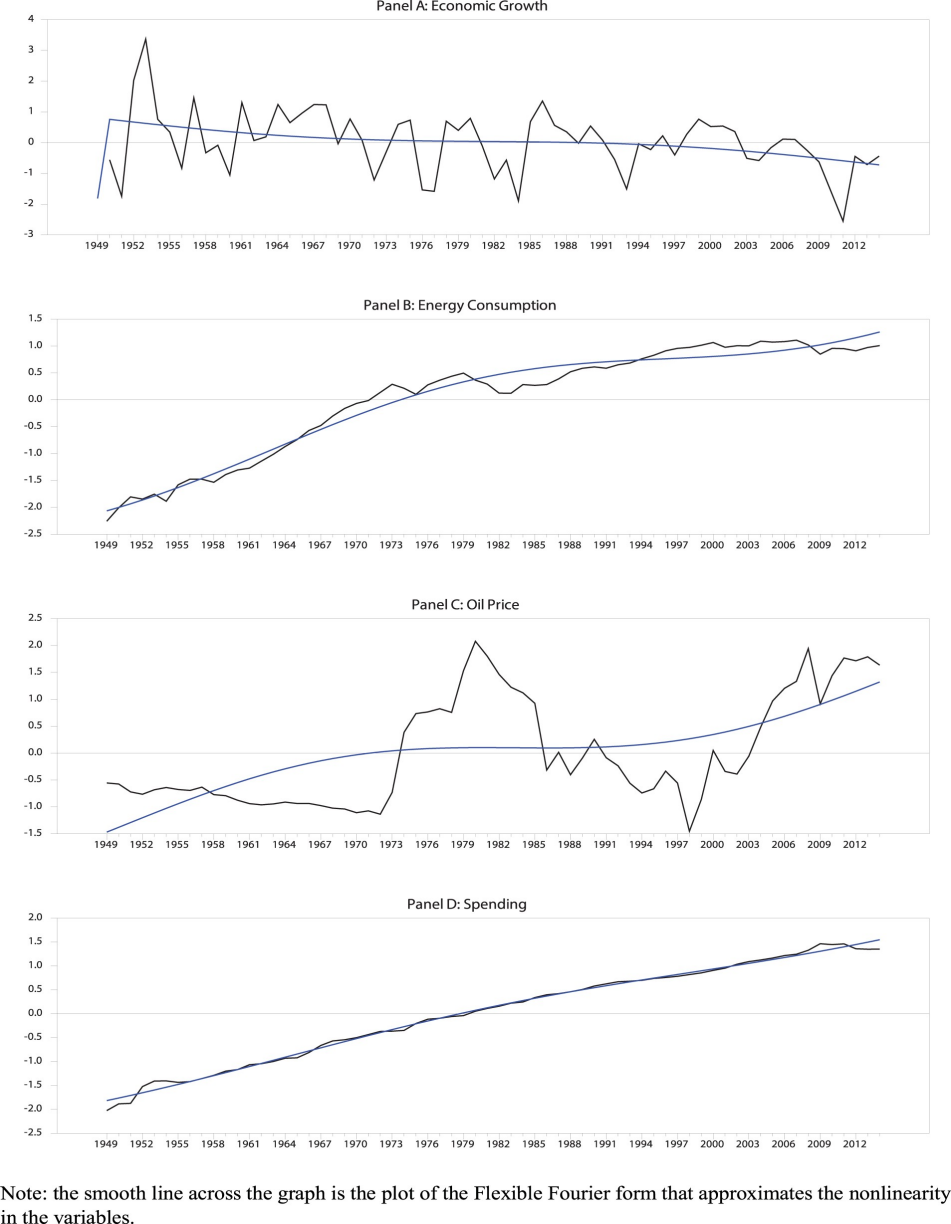
Time-trend of variables analysed.

All the data is plotted in Fig 1. Alongside the graphs of the variables we have the plot of the Flexible Fourier form that approximates the structural breaks in the variables. The data is measured annually and the sample covers the period from 1949 to 2014.

We conduct a unit root test on the variables chosen for this study. To this end, the LM based Flexible Fourier unit root test due to [40] is applied. The results of the test are shown in Table 3 below.

**Table 3:** Unit root tests with flexible fourier form.

Variable	*k*	*τ*_*LM*_
*GDP*_*t*_	1	-3.08
*EC*_*t*_	1	-3.99*
*OP*_*t*_	1	-4.01*
*SPE*_*t*_	1	-4.17**
Δ*GDP*_*t*_	1	-5.60***

***, ** and * denote rejection of the null hypothesis of a unit root at the 1%, 5% and 10% significance levels respectively. *k* representes the frequency selected for approximation. The symbol Δ is the difference operator that transforms the variable to growth from.

From the table we find that the null hypothesis of a unit root in all the variables can be rejected at least at the 10% significance level, except for the variable *GDP*_*t*_. The variables *EC*_*t*_ energy consumption, *OP*_*t*_ oil price and *SPE*_*t*_ government spending are all found to be stationary I(0) processes. Since *GDP*_*t*_ is found to contain a unit root, the variable is differenced to be made stationary. The variable Δ*GDP*_*t*_ is therefore enters the FFF-VAR model in differenced logarithmic form, describing economic growth, and hereafter labelled as *GROWTH*_*t*_

Table 4 presentes the results of the nonlinear causality tests using the FFF-VAR model. The first column lists the possible causality relations, while the second column provides the associated p-values to test the null of Granger non-causality.

**Table 4:** Test for granger causality using the FFF-VAR.

Null Hypothesis	p—values
*EC*_*t*_ ↛ *GROWTH*_*t*_	0.011
*GROWTH*_*t*_ ↛ *EC*_*t*_	0.344
*OP*_*t*_ ↛ *GROWTH*_*t*_	0.370
*GROWTH*_*t*_ ↛ *OP*_*t*_	0.757
*SPE*_*t*_ ↛ *GROWTH*_*t*_	0.115
*GROWTH*_*t*_ ↛ *SPE*_*t*_	0.933
*OP*_*t*_ ↛ *EC*_*t*_	0.006
*EC*_*t*_ ↛ *OP*_*t*_	0.049
*SPE*_*t*_ ↛ *EC*_*t*_	0.598
*EC*_*t*_ ↛ *SPE*_*t*_	0.193
*SPE*_*t*_ ↛ *OP*_*t*_	0.806
*OP*_*t*_ ↛ *SPE*_*t*_	0.227

The results show that the null hypothesis of Granger non-causality cannot be rejected for almost all possible pairs except for three cases. We can conclude energy consumption Granger causes economic growth; oil prices Granger cause energy consumption and alternatively, energy consumption Granger causes oil prices. This implies a feedback effect between oil prices and energy consumption. The nonlinear causality test provides evidence supporting the *growth hypothesis*, meaning that policies aimed at limiting energy consumption will in turn diminish economic growth. It is also interesting to note that in the nonlinear framework bidirectional causality is detected between energy consumption and oil prices. The fact that the price of oil causes energy consumption, underlies the fact that the US has not yet managed to divest from one of the most polluting fossil fuels. Also, what we find is that there are no changes in public spending to address the effects of an increase in energy consumption in the case of the US economy.

To test whether the FFF-VAR is a better fit to the data than the standard linear VAR, we conduct an F-test on the trigonometric terms, or in other words, we conduct a test for linearity. We set up the null hypothesis that the trigonometric terms are equal to zero. The results of the test are given in Table 5 below:

**Table 5:** F-test for flexible fourier form.

Variables	F-test [p-value]
*GROWTH*_*t*_	2.07 [0.05]*
*EC*_*t*_	9.97 [0.00]**
*OP*_*t*_	3.96 [0.00]**
*SPE*_*t*_	12.12 [0.00]**

**, and * denote rejection of the null hypothesis of linearity at the 5% and 1% significance levels rspectively.

The results show that we can reject the null hypothesis of the trigonometric variable exclusión test. This implies that the trigonometric terms are significant and they do approximate the small but several breaks that may exist in the data. Based on these results we can therefore conclude that the FFF-VAR provides a better fit to the data.

Given the results of the FFF-VAR we proceed to compare the causality results with those of a linear VAR. To determine how our results would compare if we had ignored the possibility of multiple structural breaks that could be gradual, we conduct a bootstrap versión of the [41] tests by implementing a procedure due to [42]. This model, in contrast to the FFF-VAR, is linear and takes in to account the order of integration of the variables. Accordingly, as a prelude to the Granger causality tests in a linear VAR framework, we conduct a comprehensive set of unit root tests to determine the order of integration of the variables. The results are shown in Table 6 below:

**Table 6:** Unit root tests.

	ADF		ADF-GLS	MZt	KPSS
	Levels	Differences			
*GDP*_*t*_	-1.78	-5.61**	-1.37	-1.32	0.22**
*EC*_*t*_	-1.29	-6.83**	-0.56	-0.58	0.23**
*OP*_*t*_	-1.89	-7.59**	-1.89	-1.79	0.07
*SPE*_*t*_	-2.01	-6.90**	-0.88	-0.61	0.30**

** denote rejection of the null at the 1% and 5% significance levels respectively.

In the first two columns we conduct the standard ADF tests in levels and differences. We cannot reject the unit root null in levels, but we can reject in first differences, thereby concluding that the data is I(1). It is well known that the ADF tests suffer from low power and accordingly we conduct a battery of unit root tests being the GLS detrended ADF tests (ADF-GLS) due to [43], M-type test due to [44] and the KPSS unit root test where the null is of stationary against the alternative of non-stationarity based on the procedure of [45]. The results broadly conclude that the data is I(1). The only exception is that of oil prices (*OP*_*t*_) where we cannot reject the null of stationarity using the KPSS test. However, in general, we can conclude that all the variables are I(1). We also carried out panel unit root tests, due to [46] and [47] for a common unit root, as well as [48] and [49] for individual unit roots. The results are the same. The results are not reported for brevity, but are available from the authors on request.

The results of no-break unit root tests stand in stark contrast to the unit root tests using flexible fourier form to approximate smooth breaks. This is not surprising as we know the unit root tests have low power to reject the null, especially in the case where structural breaks are present in the data (e.g. [50]). This is also true when there are multiple breaks in the data and the breaks can be gradual [40]. Our results shown in Table 3 where we can reject the unit root null using the Enders and Lee (ibid) method, underscores that we are using more powerful tests where we reject the unit root null taking into account the unknown nature of breaks. The results emphasise that ignoring the possible presence of multiple and gradual structural breaks in the data can lead to under-rejection of the unit root null hypothesis.

Nonetheless, based on our finding that the variables are I(1) in this particular linear form case, we proceed to carry out the [42] procedure to tests for Granger causality (We thank an anonymous referee for this comment). This procedure extends the [41] methodology. Since the [41] procedure includes non-stationary I(1) variables in the VAR making adjustments to the chosen lag length, the variables appear in the VAR in level form. The results are in Table 7 below:

**Table 7:** Bootstrapped granger causality tests.

		Bootstrapped Critical Values		
Null Hypothesis	MWALD	1% Crit. Val.	5% Crit. Val.	10% Crit. Val.
*EC*_*t*_ ↛ *GDP*_*t*_	0.139	7.246	3.972	2.753
*GDP*_*t*_ ↛ *EC*_*t*_	0.999	7.249	4.325	2.783
*OP*_*t*_ ↛ *GDP*_*t*_	0.070	8.703	4.438	2.776
*GDP*_*t*_ ↛ *OP*_*t*_	0.288	6.435	4.121	2.971
*SPE*_*t*_ ↛ GDP	0.703	7.045	4.301	2.722
*GDP*_*t*_ ↛ *SPE*_*t*_	0.001	7.228	3.854	2.585
*OP*_*t*_ ↛ *EC*_*t*_	10.34***	7.339	4.294	2.958
*EC*_*t*_ ↛ *OP*_*t*_	0.396	7.568	4.118	3.024
SPE ↛ *EC*_*t*_	0.385	6.942	4.087	2.752
*EC*_*t*_ ↛ *SPE*_*t*_	0.023	6.763	3.747	2.740
*SPE*_*t*_ ↛ *OP*_*t*_	0.849	7.862	4.298	3.073
*OP*_*t*_ ↛ *SPE*_*t*_	0.204	6.703	4.065	2.758

*** denotes rejection of the null at the 1% significance level.

The modified Wald (MWALD) tests are given in the second column of Table 2 and alongside in the three adjacent columns we tabulate the boostrapped critical values at the 1%, 5% and 10% significance levels respectively. In all the possible pairwise combinations, we find the MWALD test is lower than the bootstrapped critical values, except for the null hypothesis that oil prices (*OP*_*t*_) do not Granger cause energy consumption (*EC*_*t*_). This implies that except for oil prices causing energy consumption we cannot find any evidence of causality in all the possible pairwise combinations. The results in general show fewer rejections of the null of Granger non-causality in comparison to the results we find using the FFF-VAR. Caution needs to be exercised though, as the GDP data in this case is in levels, whereas in the FFF-VAR model the GDP data was in growth form.

## Conclusions

There is extensive literature that analyzes the causality between growth and energy consumption; however, most such studies use a bivariate approach and thus face the problem of the omission of relevant variables. Such an omission could explain the inconclusive results on the relationship between energy and growth. In addition, the studies that use multivariate models in an attempt to overcome this limitation have selected the additional variables on an ad hoc basis, thus introducing bias into the results. What is more, most studies have analyzed the existence of causality between energy and growth in a linear context, despite a broad body of literature that highlights the importance of taking into account the nonlinear dynamics in the variables associated with studies on energy consumption and economic growth.

In this paper, we overcome the limitations in the literature and analyze the relationship between energy consumption and growth in a multivariate context, choosing variables according to the probability of inclusion reported in [19]. The variables used in the model are growth, energy consumption, public spending and oil prices for the US. Besides, the existence of causality between these variables is analysed using a FFF- VAR, which approximates possible structural breaks in the variables, thereby overcoming the limitations in linear studies.

The linear causality test results indicate that there is no causality between GDP and energy consumption, and therefore a prescriptive policy measure would be to reduce energy consumption in the US without affecting the country’s economic growth. This is referred to in the literature as the ‘neutrality hypothesis’. In contrast, the nonlinear model supports the so-called ‘growth hypothesis’, since causation is found running from energy consumption to growth. This implies that a reduction in energy consumption would in fact adversely affect growth. In light of the above, it can be argued that since linear tests are unable to identify certain causal relationships, they can lead to erroneous conclusions which can have consequences for economic policy. The nonlinear methodology allows us to capture causal relationships between the other variables of the system that the linear approach fails to detect. Thus, based on the results of the linear model we would be inclined to draw the conclusion that the price of oil causes energy consumption; however, the nonlinear procedure indicates more to this relationship that there is mutual causality between the two variables. To sum up, we find the FFF-VAR results depart from those of the linear VAR. Given the evidence of nonlinearity in the data, we would be inclined to rely on the results obtained from the FFF-VAR since the Fourier form has better size and power properties; in which case there is support for the growth hypothesis, rather than the neutrality hypothesis leading to a different set of policy prescriptions.
